# A Pre-Post Study on the Appropriateness and Effectiveness of a Web- and Text Messaging-Based Intervention to Reduce Problem Drinking in Emerging Adults

**DOI:** 10.2196/jmir.2755

**Published:** 2013-09-02

**Authors:** Severin Haug, Michael P Schaub, Vigeli Venzin, Christian Meyer, Ulrich John, Gerhard Gmel

**Affiliations:** ^1^Swiss Research Institute for Public Health and Addiction at Zurich UniversityZurichSwitzerland; ^2^Cantonal Office for Secondary EducationZurichSwitzerland; ^3^Institute of Social Medicine and PreventionUniversity Medicine GreifswaldGreifswaldGermany; ^4^Addiction SwitzerlandLausanneSwitzerland

**Keywords:** alcohol intervention, problem drinking, young people, text messaging, Internet

## Abstract

**Background:**

Problem drinking, particularly risky single-occasion drinking (RSOD), also called “binge drinking”, is widespread among adolescents and young adults in most Western countries. Few studies have tested the effectiveness of interventions to reduce RSOD in young people with heterogeneous and particularly lower educational background.

**Objective:**

To test the appropriateness and initial effectiveness of a combined, individually tailored Web- and text messaging (SMS)–based intervention program to reduce problem drinking in vocational school students.

**Methods:**

The fully automated program provided: (1) online feedback about an individual’s drinking pattern compared to the drinking norms of an age- and gender-specific reference group, and (2) recurrent individualized SMS messages over a time period of 3 months. Generalized Estimating Equation (GEE) analyses were used to investigate the longitudinal courses of the following outcomes over the study period of 3 months: RSOD, alcohol-related problems, mean number of standard drinks per week, and maximum number of standard drinks on an occasion.

**Results:**

The program was tested in 36 school classes at 7 vocational schools in Switzerland. Regardless of their drinking behavior, 477 vocational school students who owned a mobile phone were invited to participate in the program. Of these, 364 (76.3%) participated in the program. During the intervention period, 23 out of 364 (6.3%) persons unsubscribed from participating in the program. The GEE analyses revealed decreases in the percentage of persons with RSOD from baseline (75.5%, 210/278) to follow-up assessment (67.6%, 188/278, *P*<.001), in the percentage of persons with alcohol-related problems (20.4%, 57/280 to 14.3%, 40/280, *P*=.009), and in the mean number of standard drinks per week: 13.4 (SD 15.3) to 11.3 (SD 14.0), *P*=.002. They also revealed a trend toward a decrease in the mean of the maximum number of drinks consumed on an occasion: 11.3 (SD 10.3) to 10.5 (SD 10.3), *P*=.08.

**Conclusions:**

The results show high acceptance and promising effectiveness of this interventional approach, which could be easily and economically implemented within school classes.

## Introduction

Alcohol use is a major cause of the disease burden in most countries of the world [1]. In Europe, alcohol is responsible for 12% of male and 2% of female premature death and disability [2]. Problem drinking is associated with multiple social and interpersonal problems [2,3]. Indicators of problem drinking are: (1) a daily average consumption of 30 g or more of pure alcohol for men and 20 g or more for women [4], and (2) risky single-occasion drinking (RSOD) (also called “binge drinking”), defined as drinking 5 or more drinks on one occasion for men and 4 or more drinks for women [5]. The prevalence rates of RSOD are particularly high in adolescence and young adulthood and are higher among men than among women [6].

Studies testing the efficacy of interventions to reduce problem drinking in young people have been conducted predominantly in the United States and have been targeted toward college or university students [7]. Within this target group, individual interventions using motivational interviewing [8] or personalized normative feedback, based on the social norms approach [9], showed promising findings and resulted in lower alcohol consumption and fewer alcohol-related problems [10,11].

Social norm interventions provide information about the actual drinking norm in a reference group. They typically include an individualized drinking profile with the quantity of alcohol consumed in relation to peers, the money spent on alcohol, the calorie intake, and the individual risk patterns of alcohol-related negative consequences. In university and college students, normative feedback interventions delivered using the Web or computer, reduced drinking quantity, drinking frequency, and binge drinking in the short- and medium-term [12].

Computer-tailored interventions based on the social norms approach are also promising for the reduction of problem drinking in populations with lower educational backgrounds. However, it should be noted that the efficacy of tailored messages depends on the individual’s ability and motivation to process information. The Elaboration Likelihood Model [13] posits that the degree to which individuals are motivated and able to process a persuasive message (Need for Cognition or NFC) determines the care with which the central merits of a message will be considered and evaluated. Individuals with low NFC pay more attention to the source of the arguments (eg, celebrities, credible authorities, experts), the ease with which they can be processed (eg, presented pictorially versus verbally), and the number of arguments presented, to process information. Although NFC is thought to reflect a cognitive motivation rather than an intellectual ability, it is positively correlated with educational level [14]. Considering individuals’ NFCs in the context of health behavior, interventions could be crucial for improving their outcomes [15-17].

Social norm interventions for reducing problem drinking typically consist of a single intervention session in which participants receive tailored Web or printed feedback. Due to their length of up to 7-8 pages of text and graphics, these interventions are primarily matched with persons of higher educational levels and higher NFCs. An approach that might be more effective for individuals with lower educational levels and that might also be more persuasive if persons are processing information on the peripheral route is to provide shorter, more recurrent feedback messages.

Text messaging (short message service, SMS), which is available primarily via mobile phones, provides a suitable technology to deliver short and repeated feedback messages. This service allows a cost-effective instantaneous delivery of short messages directly to individuals at any time and place. In the field of alcohol prevention, SMS particularly allows the delivery of individualized messages at times when young people typically drink alcohol [18]. In Switzerland, as in most other developed countries, nearly all adolescents (98%) between the ages of 12 and 19 own a mobile phone, and SMS is the most commonly used mobile phone application [19].

SMS is increasingly being applied for behavior change interventions, particularly in smoking cessation and diabetes self-management [20,21]. For alcohol treatment, 2 pilot studies based on relatively small sample sizes are available. Suffoletto et al [22] reported fewer heavy drinking days and fewer drinks per drinking day in 15 young adults reporting harmful alcohol use receiving SMSs up to 3 months after emergency treatment. In a study using twice daily supportive text messages (n=26) or a thank you text message (n=28) every 2 weeks for 3 months, co-morbid depressive and alcohol-dependent patients reported lower depression scores and a trend for higher cumulative abstinence duration [23].

Vocational school students are typically characterized by heterogeneous educational levels, including a significant proportion with little or no educational attainment, and high prevalence rates of hazardous drinking [24]. The objectives of the present study were (1) to test the appropriateness of a combined Web- and SMS-based intervention program for the reduction of problem drinking in vocational school students, and (2) to provide an initial test of its effectiveness.

## Methods

### Setting

In most European countries, vocational schools are post-secondary public schools that are analogous to American community colleges. They are part of the dual educational system that combines apprenticeships in a business context and vocational training in a school context. Vocational schools provide general education and specific skills for a particular profession.

Based on data from the Swiss Federal Statistical Office, approximately half of all adolescents ages 16 to 19 currently attend vocational schools [25], with the highest proportions among adolescents ages 17 (males: 63%, females: 48%) and 18 (males: 63%, females: 49%).

### Design and Procedure

A longitudinal pre-post study design was used to test the initial effectiveness of the program. Furthermore, differences in the longitudinal courses of the main outcome criteria between program participants and nonparticipants were explored.

Directors or contact teachers for addiction prevention from all 23 vocational schools in the Swiss canton of Zurich were invited to participate through some of their classes in a study testing the effectiveness of a Web- and text messaging-based program to reduce problem drinking. Of these 23 schools, 7 vocational schools with a total of 36 school classes agreed to participate in the study. All vocational school students in the participating school classes were invited by externally trained staff to participate in an online health survey during a regular school lesson reserved for health education. To decrease reporting bias, the study assistants did not provide further information about the purpose of the study before the screening assessment was completed. The screening assessments were conducted between April 2012 and September 2012. At the time of the assessment, 490 students were present in the school classes, of whom 488 (99.6%) agreed to participate. The online screening included the assessment of demographic data, alcohol consumption, weekly physical activity, smoking status, and ownership of a mobile phone.

The inclusion criterion for program participation was ownership of a mobile phone. A total of 477 of the 488 participants (97.7%) from the screening assessment owned a mobile phone. Subsequently, eligible persons were informed about the aim of the program, assessments, reimbursement, and data protection. Study and program information was provided online and in paper form by the study assistants. Eligible persons were informed that they could unsubscribe from program participation at any time simply by sending an SMS expressing their request to withdraw from the program. Additionally, they were informed that program participants would take part in a draw for 10 vouchers worth €50. Eligible persons could then decide whether to participate in the program or not. After providing informed consent online, all program participants were invited to choose a username and to provide their mobile phone number. Furthermore, additional alcohol-related variables were assessed.

Follow-up assessments after 3 months were conducted in the participating school classes during regular school lessons using paper-and-pencil questionnaires. All vocational school students present in the school classes at the follow-up assessments (also students who were not eligible for participation in the program and nonparticipants) were invited to fill in a questionnaire.

The study protocol was approved by the local Ethics Committee of the Canton of Zurich, Switzerland. The study was executed in compliance with the Declaration of Helsinki.

### Intervention

The fully automated program was based on a LAMPP-system (Linux system, Apache server, MySQL-database, PHP-programming language) and included an expert system that generated individually tailored online feedback and text messages. The interventional content was based on effective social norms intervention programs developed primarily for college and university students in the United States and Canada [26,27] that had been modified for the target group of German-speaking adolescents in Switzerland, aged 16-20 with different educational backgrounds.

The program, Alk-Check, automatically generated individually tailored online feedback and SMS messages using data from a comprehensive online assessment. The online assessment tool collected demographic information and information on alcohol consumption, drinking behavior (eg, typical drinking days and times), and alcohol-related problems. Age- and gender-specific norms for alcohol consumption were derived from a previous study [28] that assessed heavy drinking occasions, alcohol volume, and the maximum number of drinks on a single occasion among 973 vocational and secondary school students in the Canton of Zurich, Switzerland.

After completing the online assessment, individually tailored online feedback was provided. The online feedback was tailored according to the individual values on the following 4 baseline variables: gender, age, number of standard drinks in a typical week, and frequency of RSOD occasions in the last 30 days. The feedback included graphical and textual information concerning (1) drinks per week in relation to the age and gender-specific reference group, (2) financial costs of drinking, (3) calories consumed with alcoholic drinks, and (4) number of heavy drinking occasions in relation to the age and gender-specific reference group. The online feedback could be printed and sent by mail to the participants’ email accounts.

On the first level, the content and number of text messages were tailored according to baseline drinking patterns. Participants were assigned to one of three risk groups (derived from [4,5]), based on their baseline drinking patterns: (1) “Non-Risk”: No RSOD occasion during the last 30 days and <18 (12 for females) standard drinks in a typical week, (2) “Low-Risk”: 1 or 2 RSOD occasions during the last 30 days or no RSOD occasions during the last 30 days, and ≥18 (12 for females) standard drinks in a typical week, and (3) “High Risk”: >2 RSOD occasions during the last 30 days.

On the second level, the content of the text messages was tailored according to the individual values on the following baseline variables: gender, motivation for reduced alcohol consumption, alcohol-related problems, typical drinking day and time, number of standard drinks in a typical week, and maximum number of drinks on a single occasion during the last 30 days.

Participants from all risk groups received text messages for a period of 12 weeks. Participants of the non-risk group received one weekly text message providing information from the following content categories: (1) drinking and body weight/fitness, (2) resisting peer pressure, (3) pros of sensible drinking, and (4) motivation to maintain sensible drinking.

Participants of the low-risk group received one weekly text message providing information from the following content categories: (1-3), (5) motivation for sensible drinking, (6) alcohol-related problems, (7) maximum number of drinks on a single occasion and related risks, (8) risks of binge drinking, and (9) importance of reducing alcohol consumption. Additionally, they received biweekly text messages sent on the individually indicated typical drinking day and time. The latter messages specifically focused on strategies to reduce alcohol consumption and to motivate them toward sensible drinking practices. Participants of the high-risk group received one weekly text message providing information from the content categories: (1-3), (5-9), and (10) local outpatient services for alcohol counseling. They also received the additional biweekly text messages sent on the individually indicated typical drinking day and time that focused on strategies to reduce alcohol consumption and to motivate them to adopt sensible drinking practices. Examples of these text messages are shown in Table 1.

Before the study, a prototype of this program was tested and evaluated in 3 focus groups. Within these focus groups, vocational school adolescents aged between 16 and 20 years evaluated the program flow, the layout and content of the online assessment and feedback, and the content of the text messages. The optimizations that resulted from these focus groups were integrated in the final program version.

### Measures and Outcome Criteria

The screening assessment included the following demographic variables: gender, age, education, and migration background. Common Swiss levels of educational attainment were assessed: (1) none, (2) secondary school, (3) extended secondary school, and (4) technical or high school. We assessed the country of birth of both parents of the vocational school students to identify a potential migration background. Based on this information, persons were assigned to one of the following categories: (1) persons with neither parent born outside Switzerland, (2) persons with one parent born outside Switzerland, and (3) persons with both parents born outside Switzerland.

Tobacco smoking was assessed using the question, “Do you currently smoke cigarettes or did you smoke in the past?” with the following response options: (1) I smoke cigarettes daily, (2) I smoke cigarettes occasionally, but not daily, (3) I smoked cigarettes in the past, but I do not smoke anymore, and (4) I have never smoked cigarettes or have smoked fewer than 100 cigarettes in my life. Current daily and occasional smokers (categories 1 and 2) were considered smokers. Self-reported moderate to vigorous physical activity was measured by a question derived from the Health Behavior in School Aged Children (HBSC) study [29]: “Outside school: How many hours a week do you exercise or participate in sports that make you sweat or out of breath?”.

The following 3 alcohol-related variables were included in the screening assessment: (1) frequency of RSOD occasions in the last 30 days (“How often did you have 5 [4 for females] or more drinks on one occasion in the last 30 days?” with the response categories “never”, “1-2 times”, “3-4 times”, “5-6 times”, “7-8 times”, “9-10 times”, “11-12 times”, “more than 12 times”); (2) quantity of alcohol consumption, as assessed by a 7-day drinking calendar similar to the Daily Drinking Questionnaire (DDQ) [30], for which participants were asked to think about a typical week in the past month and, for each day, to record the number of standard drinks they typically consumed on that day; and (3) the maximum number of drinks consumed on a single occasion in the last 30 days.

In the program, we also assessed alcohol-related problems and the motivation to reduce their alcohol consumption among participants. Alcohol-related problems were assessed by a questionnaire derived from the European School Survey Project on Alcohol and Other Drugs (ESPAD) [31]. The participants were asked about the number of occasions during the last 3 months when they had experienced problems related to their alcohol use. Ten problems are listed in the questionnaire, which could be grouped into 4 categories: (1) individual problems, (2) relational problems, (3) sexual problems, and (4) delinquency problems. The importance of reducing alcohol consumption was assessed by the question, “How important is it for you to modify your alcohol consumption and to drink less?” with the response categories “very important”, “rather important”, “rather unimportant”, and “very unimportant”.

To obtain the number of program participants who unsubscribed from the program (program attrition), we analyzed the log files of the SMS system in which all incoming and outgoing text messages were recorded.

At follow-up, we also assessed an aspect of the usage of the SMS messages by asking the participants whether they (1) read through the SMS feedback messages thoroughly, (2) took only a short look at the feedback messages, or (3) did not read the feedback messages. Using a yes/no question, we evaluated whether the times when participants received the SMS messages were appropriate. We assessed whether the number of received SMS messages was appropriate or whether the participants would have preferred less or more SMS messages. The program participants also indicated their approval of certain statements concerning different aspects of the SMS messages and the online feedback (comprehensibility, content, degree of tailoring), using the response categories “rather yes” and “rather no”.

Outcome criteria for the test of effectiveness of the intervention were: (1) RSOD in the last 30 days (yes/no), (2) frequent RSOD in the last 30 days (0-2 RSOD occasions vs >2 RSOD occasions), (3) number of standard drinks in a typical week, (4) maximum number of drinks on an occasion in the last 30 days, and (5) alcohol-related problems in the last 3 months (yes/no).

### Data Analyses

To test for baseline differences between program participants and nonparticipants, chi-square tests for categorical variables and Mann-Whitney U-tests for continuous or ordinal variables were used. For the attrition analysis (program participants lost to follow-up), we also used chi-square tests for categorical variables and Mann-Whitney U-tests for continuous or ordinal variables.

We used Generalized Estimating Equation (GEE) analyses to investigate the longitudinal course of the outcome criteria over the study period of 3 months. GEE is a repeated-measures regression model that takes into account the correlation of the repeated measures within a person [32]. It is a powerful and versatile procedure for analyzing longitudinal data under minimal assumptions about time dependence and allowed us to use all available longitudinal data, regardless of single missing values at follow-up.

We used logistic GEE models for the binary outcome variables “RSOD” and “alcohol-related problems”, as well as GEE models for the count data of the variables “number of standard drinks in a typical week” and “maximum number of drinks on a single occasion”. Each GEE-model included the examined time variable (baseline vs follow-up assessment) as a predictor and an outcome variable as dependent variable.

Given the clustered nature of the data (students within school classes), we computed robust variance estimators for all GEE analyses. An alpha level of 0.05 (2-tailed) was chosen for all statistical tests in this study. All analyses were performed using the Stata software package, version 10.

Beyond the statistical examination of the longitudinal course of the outcome variables for program participants, we graphically explored the longitudinal course of the outcome variables for non-program participants in contrast to program participants. Due to a lack of statistical power, we did not use statistical tests for this comparison.

**Table 1:** Sample text messages from different risk groups and content categories.

Risk Group	Content Category	Text Message
Non-Risk	Drinking and body weight/fitness	Hi Peter. Alcohol is rich in calories, slows down the body’s burning of fat and increases one’s appetite. In short: drinking alcohol regularly makes you overweight in the long term. It’s great that you don’t drink alcohol at all!
Non-Risk	Resisting peer pressure	Hi Sarah. You are not just a follower who drinks alcohol to fit in. Awesome! This shows strength of character and can even impress others. Only do what you think is right.
Non-Risk	Pros of sensible drinking	Hey. Even with a blood alcohol content of only 0.03% (eg, 1 to 2 beers), you have an increased risk of accidents. Whether by walking, riding your bike or driving your car, without alcohol in the blood you are always safer on the road. Way to go!
Low-Risk	Importance of reducing alcohol consumption	Hi. You would like to drink less alcohol. That’s a smart decision for you! If you consume less alcohol, you will feel better and have more energy the next day.
Low-Risk	Alcohol-related problems	Hello Lucy, due to consumption of alcohol, you’ve had problems with your parents. That’s not necessary! Keep in mind that you can avoid these problems by drinking less or no alcohol at all!
High-Risk	Maximum number of drinks on a single occasion and related risks	Hey Mike. You recently had 14 drinks on one occasion. Your blood alcohol concentration was about 0.34% that time. With that amount of alcohol in your blood you can experience unconsciousness, loss of memory, shallow breathing, a reduction of body temperature and loss of reflexes. Watch out!
High-Risk	Strategies to reduce alcohol consumption and to motivate for sensible drinking	Hey. It’s good for your body to have soft drinks every now and then! Non-alcoholic drinks provide your body with important minerals and are a good, thirst-quenching alternative. By drinking them you can prevent yourself from getting drunk as quickly.
High-Risk	Local outpatient services for alcohol counseling	Hi Robin. Are you concerned about your own alcohol intake or that of a friend? Talking to someone about it can be really helpful. The website www.alcocheck.ch can offer you support. Write an email to info@alcocheck.ch or call 043 444 77.

## Results

### Study Participants

Of the 477 persons who owned a mobile phone and were therefore eligible for study participation, 364 (76.3%) registered for program participation. Table 2 presents the demographic, health, and alcohol-related characteristics of program participants and nonparticipants. Program participants differed from nonparticipants with respect to the baseline variables “educational attainment” and “maximum number of drinks on an occasion in the last 30 days”. Program participants had a lower level of educational attainment (*U*=17402.0, *P*<.001) and had a higher maximum number of drinks on a single occasion (*U*=17958.5, *P*=.04).

After 3 months, 367 of the 477 (76.9%) students who were eligible for program participation completed follow-up assessment: program participants 280/364 (76.9%); nonparticipants 87/113 (77.0%). The attrition analysis showed that persons dropping out were significantly more likely to be smokers (χ^2^=6.2, *P*=.01) and to have more frequent RSOD within the last month (*U*=17297.5, *P*=.02).

### Appropriateness of the Intervention

#### Program Attrition

During the program, which lasted for 3 months, 23 out of the 364 (6.3%) program participants unsubscribed from participating in the program.

#### Program Use and Evaluation

Out of the 280 program participants who could be reached for follow-up assessment, we obtained data concerning the program use and evaluation from 234 to 269 persons, depending on the respective variable. These different frequencies are due to missing data, inconsistent data, or variables that were only assessed in some persons depending on their previous answers.

Of the program participants, 254 out of 269 persons (94.4%) with valid data indicated that they regularly received the SMS messages. Of the 249 persons with valid data, 124 (49.8%) indicated that they “read the SMS messages thoroughly”; 111 persons (44.6%) reported that they “took a short look at the feedback messages”; and 14 persons (5.6%) chose the predefined response category, “I did not read the feedback messages”.

The time when participants received the SMS messages was rated as appropriate by 75.4% of the program participants (196/260). The number of received SMS messages was rated as appropriate by 57.5% (149/259); 35.5% (92/259) would have preferred fewer; and 6.9% (18/259) would have preferred more SMS messages. Table 3 presents additional evaluations of the tailored online feedback and of the SMS messages by the program participants.

**Table 2:** Baseline characteristics of program participants and nonparticipants; values are numbers (%), unless stated otherwise.

		Program participants (n=364)	Non-participants (n=113)
Female gender		89 (24.5%)	22 (19.5%)
**Age in years, mean (SD)**		18.0 (2.4)	17.8 (1.7)
	15-16 years	73 (20.1%)	20 (17.7%)
	17-18 years	194 (53.3%)	64 (56.6%)
	19-20 years	72 (19.8%)	23 (20.4%)
	21 years or older	25 (6.9%)	6 (5.3%)
**Immigration background**			
	No immigration background	185 (50.8%)	45 (39.8%)
	One parent born outside Switzerland	69 (19.0%)	22 (19.5%)
	Both parents born outside Switzerland	110 (30.2%)	46 (40.7%)
**Educational attainment**			
	None	18 (4.9%)	3 (2.7%)
	Secondary school	300 (82.4%)	80 (70.8%)
	Extended secondary school	39 (10.7%)	24 (21.2%)
	Technical or high school	7 (1.9%)	6 (5.3%)
**Tobacco smoking**			
	Never smokers or recent quitters	171 (47.0%)	65 (57.5%)
	Current daily or occasional smokers	193 (53.0%)	48 (42.5%)
Hours of extracurricular moderate to vigorous physical activity per week, M (SD)		4.5 (4.6)	4.8 (3.9)
**Frequency of risky single-occasion drinking in the last 30 days**			
	Never	85 (23.4%)	36 (31.9%)
	1-2 times	106 (29.1%)	29 (25.7%)
	3-4 times	71 (19.5%)	23 (20.4%)
	5-6 times	48 (13.2%)	5 (4.4%)
	7-8 times	20 (5.5%)	7 (6.2%)
	9-10 times	14 (3.8%)	5 (4.4%)
	11-12 times	6 (1.6%)	1 (0.9%)
	More than 12 times	14 (3.8%)	7 (6.2%)
Number of standard drinks in a typical week, mean (SD)		14.1 (16.1)	11.3 (14.4)
Maximum number of drinks on an occasion in the last 30 days, mean (SD)		11.6 (10.8)	10.0 (11.3)
One or more alcohol-related problems in the last 3 months		80 (22.0%)	
**Importance of reducing alcohol consumption**			
	Very unimportant	164 (45.1%)	
	Rather unimportant	121 (33.2%)	
	Rather important	48 (13.2%)	
	Very important	31 (8.5%)	

**Table 3:** Evaluation of online feedback and text messages by program participants; values are numbers (%).

		Rather Yes, n (%)	Rather No, n (%)
**The online feedback was…**			
	comprehensible (n=265 )	250 (94.3)	15 (5.7)
	interesting (n=258)	182 (70.5)	76 (29.5)
	individually tailored for me (n=257)	127 (49.4)	130 (50.6)
**The text messages were…**			
	comprehensible (n=240)	232 (96.7)	8 (3.3)
	helpful (n=235)	71 (30.2)	164 (69.8)
	individually tailored for me (n=234)	79 (33.8)	155 (66.2)

### Program Effectiveness

#### Risky Single-Occasion Drinking (RSOD)

**Figure 1:**
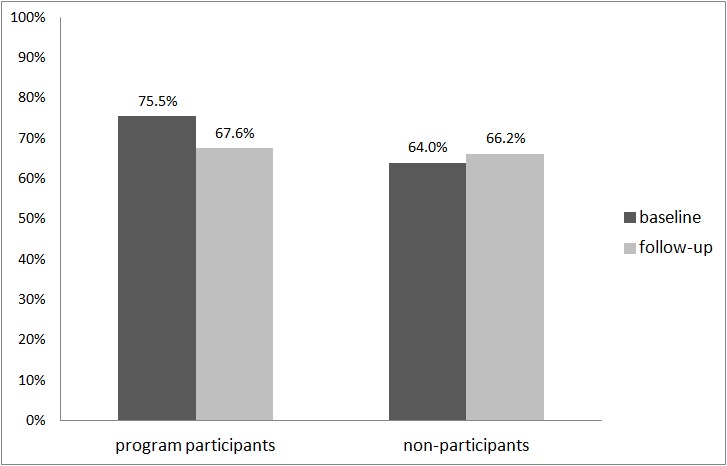
Percentage of persons with at least one RSOD occasion in the last month (program participants: n=278; nonparticipants: n=86).

The GEE analyses revealed a statistically significant decrease in the percentage of persons with at least one RSOD occasion in the last month from the baseline assessment to the follow-up assessment (OR 0.66, 95% CI 0.53-0.83, *P*<.001). Considering only program participants with appropriate follow-up data, the percentage of program participants with at least one RSOD occasion in the last month was 75.5% (210/278) at baseline and 67.6% (188/278) at follow-up (Figure 1).

**Figure 2:**
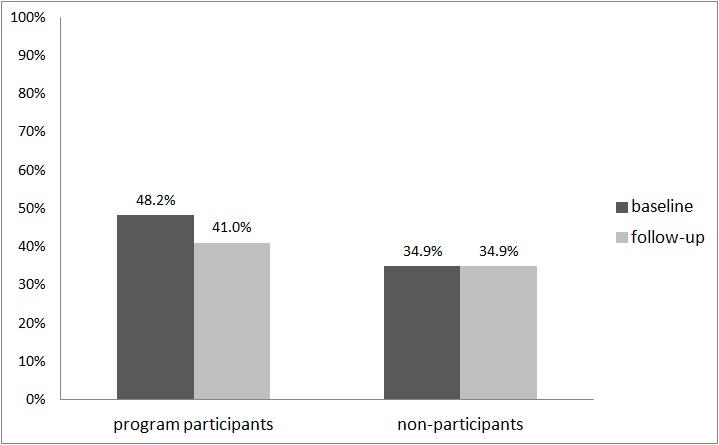
Percentage of persons with more than two RSOD occasions in the last month (program participants: n=278; nonparticipants: n=86).

The GEE analyses also revealed a statistically significant decrease in the percentage of persons with more than two RSOD occasions in the last month from the baseline assessment to the follow-up assessment (OR 0.76, 95% CI 0.61-0.94, *P*=.01). Considering only program participants with follow-up data, the percentage of program participants with more than two RSOD occasions in the last month was 48.2% (134/278) at baseline and 41.0% (114/278) at follow-up (Figure 2).

#### Number of Standard Drinks in a Typical Week

**Figure 3:**
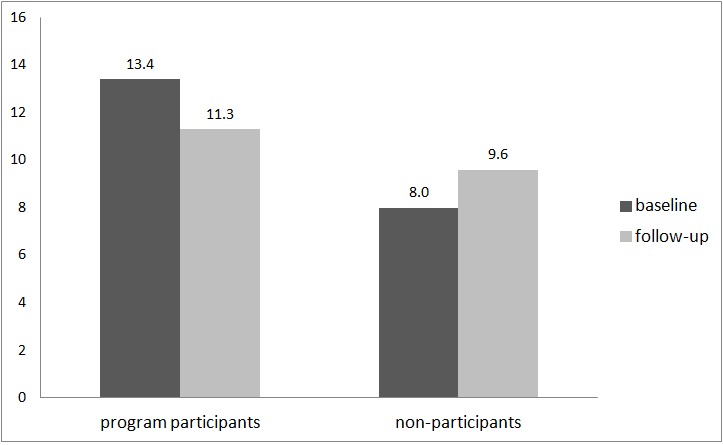
Mean number of standard drinks in a typical week (program participants: n=247; nonparticipants: n=79).

The GEE analyses revealed a statistically significant decrease in the number of standard drinks in a typical week from the baseline assessment to the follow-up assessment (IRR 0.83, 95% CI 0.74-0.93, *P=*.002). Considering only program participants with follow-up data, the mean number of standard drinks in a typical week was 13.4 (SD 15.3) at baseline and 11.3 (SD 14.0) at follow-up (Figure 3).

#### Maximum Number of Drinks on an Occasion

**Figure 4:**
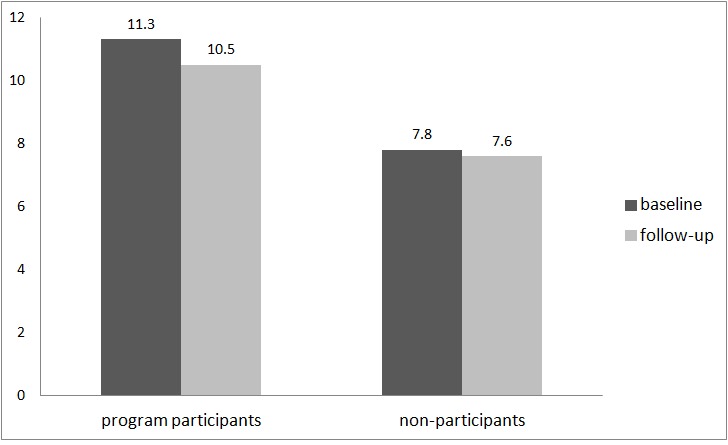
Maximum number of drinks on an occasion (program participants: n=275; nonparticipants: n=83).

An effect close to reaching statistical significance was observed on the decrease in the maximum number of drinks on an occasion in the last 30 days from baseline to follow-up assessment (IRR 0.91, 95% CI 0.83-1.01, *P*=.08). Considering only program participants with follow-up data, the maximum number of drinks on an occasion was 11.3 (SD 10.3) at baseline and 10.5 (SD 10.3) at follow-up (Figure 4).

#### Alcohol-Related Problems

The GEE analyses revealed a statistically significant decrease in the percentage of persons with one or more alcohol-related problems in the last 3 months from the baseline assessment to the follow-up assessment (OR 0.60, 95% CI 0.41-0.88, *P*=.009). Considering only program participants with follow-up data, the percentage of persons with one or more alcohol-related problems in the last 3 months was 20.4% (57/280) at baseline and 14.3% (40/280) at follow-up.

## Discussion

### Principal Findings

The study revealed three main findings: (1) a large percentage of vocational school students could be reached by the program, (2) the acceptance of the program was good, and (3) the program may reduce problematic alcohol consumption in young people with heterogeneous and primarily lower educational levels.

The data of this study and other epidemiological studies [24,33] showed high prevalence rates of problem drinking in vocational school students compared to population surveys of adolescents and young adults in this age group [34]. Within this high-risk group, the proactive invitation for program participation in combination with the offer of a low-threshold intervention using the Internet and SMS allowed us to reach 3 out of 4 vocational school students (76%) for participation in the program Alk-Check. Taking into account that 4 out of 5 (78%) program participants indicated that a reduction of alcohol consumption was rather unimportant or unimportant for them, this high participation rate is of special relevance. It underlines the importance of proactive recruitment and the attractiveness of the applied communication media for this target group.

Participation in the program was relatively independent of gender, age, and immigration background, but participation rates were higher for persons with lower school educations and for persons with higher alcohol consumption. The latter finding shows that we were able to particularly reach the main target group of the intervention, namely young people with problem drinking.

The overall acceptance of the intervention was good. Nearly all program participants (94%) stayed logged in until the end of the program lasting 3 months. The SMS messages were read by almost all program participants (94%), and both the SMS messages and the online feedback were comprehensible for almost all participants (SMS: 97%, online feedback: 94%). Room for improvement was indicated in particular concerning the tailoring of the SMS messages. While nearly half of the participants (50%) rated the online feedback as individually tailored, only 34% indicated that they perceived the SMS messages as individually tailored.

The results concerning the initial effectiveness of this program derived from a pre-post investigation are promising. The data revealed a statistically significant decrease in the percentage of persons with at least one RSOD occasion in the last month from baseline assessment (76%) to follow-up assessment (68%), as well as a statistically significant decrease in the percentage of persons with more than two RSOD occasions in the last month (from 48% to 41%). Furthermore, we found statistically significant decreases in the percentages of persons with alcohol-related problems and in the mean number of standard drinks per week. These positive changes could not be observed in persons not participating in the program.

### Limitations

One limitation of this study is its lack of a control group that was derived on the basis of random assignment. Although participants were included into the study regardless of their drinking behavior, the comparison of baseline characteristics of program participants and nonparticipants indicates toward higher levels of alcohol consumption in program participants. Therefore, beyond the intervention effects, regression to the mean might have influenced the course of alcohol-related variables from baseline to follow-up. However, lack of a control group resulted in a greater proximity to prevention practices and allowed a better estimation of the participation rate in the program.

### Conclusions

This is the first study to test a combined Web- and SMS-based intervention program for the reduction of problem drinking and one of the very few studies to test an intervention reducing problem drinking in a school sample. The results of this study show appropriateness and promising effectiveness for this intervention approach of combining singular online feedback to provide comprehensive individualized data about a person’s alcohol consumption compared to an age- and gender-specific reference group and repeated individualized SMS messages encouraging sensible drinking. The intervention could be easily and economically implemented within school classes. Based on these initial positive results, testing this interventional approach within a randomized controlled trial would be reasonable.

## Abbreviations

DDQ: Daily Drinking Questionnaire
ESPAD: European School Survey Project on Alcohol and Other Drugs
GEE: Generalized Estimating Equation
HBSC: Health Behavior in School Aged Children study
NFC: Need for Cognition
RSOD: risky single-occasion drinking
SMS: short message service
